# PKN2 and Cdo interact to activate AKT and promote myoblast differentiation

**DOI:** 10.1038/cddis.2016.296

**Published:** 2016-10-20

**Authors:** Sang-Jin Lee, Jeongmi Hwang, Hyeon-Ju Jeong, Miran Yoo, Ga-Yeon Go, Jae-Rin Lee, Young-Eun Leem, Jong Woo Park, Dong-Wan Seo, Yong Kee Kim, Myong-Joon Hahn, Jeung-Whan Han, Jong-Sun Kang, Gyu-Un Bae

**Affiliations:** 1 Research Center for Cell Fate Control, College of Pharmacy, Department of Cell Therapy, Sookmyung Women's University, Seoul, Republic of Korea; 2 Department of Molecular Cell Biology, Sungkyunkwan University School of Medicine, Samsung Biomedical Research Institute, Suwon, Republic of Korea; 3 Research Center for Epigenome Regulation, School of Pharmacy, Department of Biochemistry and Molecular Biology, Sungkyunkwan University, Suwon, Republic of Korea; 4 College of Pharmacy, Department of Biochemistry, Dankook University, Cheonan, Republic of Korea

## Abstract

Skeletal myogenesis is coordinated by multiple signaling pathways that control cell adhesion/migration, survival and differentiation accompanied by muscle-specific gene expression. A cell surface protein Cdo is involved in cell contact-mediated promyogenic signals through activation of p38MAPK and AKT. Protein kinase C-related kinase 2 (PKN2/PRK2) is implicated in regulation of various biological processes, including cell migration, adhesion and death. It has been shown to interact with and inhibit AKT thereby inducing cell death. This led us to investigate the role of PKN2 in skeletal myogenesis and the crosstalk between PKN2 and Cdo. Like Cdo, PKN2 was upregulated in C2C12 myoblasts during differentiation and decreased in cells with Cdo depletion caused by shRNA or cultured on integrin-independent substratum. This decline of PKN2 levels resulted in diminished AKT activation during myoblast differentiation. Consistently, PKN2 overexpression-enhanced C2C12 myoblast differentiation, whereas PKN2-depletion impaired it, without affecting cell survival. PKN2 formed complexes with Cdo, APPL1 and AKT via its C-terminal region and this interaction appeared to be important for induction of AKT activity as well as myoblast differentiation. Furthermore, PKN2-enhanced MyoD-responsive reporter activities by mediating the recruitment of BAF60c and MyoD to the myogenin promoter. Taken together, PKN2 has a critical role in cell adhesion-mediated AKT activation during myoblast differentiation.

For efficient regeneration of damaged tissues, stem cells need to respond properly to the extracellular cues to proliferate and to facilitate the differentiation process. Skeletal muscle differentiation is a multistep process that involves cell cycle withdrawal, expression of muscle-specific genes and formation of multinucleated myofibers by cell fusion.^1^ This process is coordinated by two groups of transcription factors, the myogenic determination factors and the myocyte enhancer factor 2 (MEF2) family.^2, 3, 4^ These transcription factors are tightly regulated to ensure efficient differentiation and to maintain the differentiated state of cells.^5, 6^ Myoblast differentiation requires a specific recognition and adhesion between muscle progenitors. Several downstream signaling pathways, including p38MAPK, Rho family small GTPases and AKT are implicated in cell adhesion-mediated myogenesis.^7, 8, 9, 10^ A cell surface receptor Cdo (cell adhesion molecule-related downregulated by oncogene, also called Cdon) integrates cell contact-mediated signals from cell surface into the myogenic regulatory network.^11^ Cdo forms multiprotein complexes with other cell adhesion molecules including N-cadherin, Gas1, Boc and Neogenin and promotes myogenesis.^12, 13, 14, 15^ Cdo-depleted myoblasts show inefficient myogenic differentiation and Cdo-deficient mice display a delayed skeletal muscle development.^9, 16^ The promyogenic function of Cdo involves a coordinated activation of p38MAPK and AKT via association with scaffold proteins, JLP and Bnip-2 for Cdc42 and p38MAPK.^9, 17^ and APPL1 for AKT.^7^ Well-supported evidences have suggested that AKT signaling has important roles in myoblast differentiation^8, 18, 19^ and insulin-like growth factor (IGF)-mediated myoblast survival, which is critically activated during myogenic differentiation.^20, 21^ AKT overexpression enhances myoblast differentiation, whereas AKT inhibition by expression of a dominant-negative AKT blocks myotube formation. The suppression of myogenesis caused by PI3-kinase inhibition is rescued by the ectopic expression of a constitutively active AKT.^22^

Protein kinase C-related kinases (PKN/PRKs) are serine/threonine kinases and consist of three isoforms, PKN1, PKN2 and PKN3,^23^ which contain three tandem HR1 domains at their N-terminal region, a calcium-binding C2-like domain and a C-terminal PKC-like serine/threonine kinase domain.^24^ PKNs function as effectors of Rho GTPases in diverse cellular pathways,^24, 25, 26, 27, 28^ such as cytoskeletal organization,^25^ cell adhesion,^26^ cell cycle control^27^ as well as cell migration,^28^ PKN2 appears to regulate cell–cell adhesion,^26^ apical junction maturation in keratinocytes^29^ and migration of astrocytes.^30^ Furthermore, PKN2 can be cleaved by caspases at amino acid (AA) 700 and the resulting C-terminal fragment can interact and inhibit AKT during apoptosis in 293 and COS cells.^31^ PKN2 is expressed ubiquitously in developing embryos,^32^ although its role in myogenesis is currently unclear. Considering the proposed role of PKN2 in cytoskeletal organization and cell adhesion signaling regulated by Rho GTPases and its interaction with AKT, prompt us to assess its role in myogenesis, especially in Cdo-mediated promyogenic pathway.

Like Cdo, PKN2 was induced in differentiating C2C12 myoblasts. PKN2 was decreased in Cdo-depleted cells accompanied by diminished AKT activation. Overexpression of PKN2 in C2C12 cells enhanced myoblast differentiation, whereas PKN2-depletion led to impaired differentiation. PKN2 interacted with Cdo, APPL1 and AKT via its C-terminal region, and this interaction appeared to be important for AKT activation in myoblast differentiation thereby positively regulating myoblast differentiation.

## Results

### PKN2 was upregulated during myoblast differentiation and decreased in Cdo-depleted myoblasts

**Figure 1:**
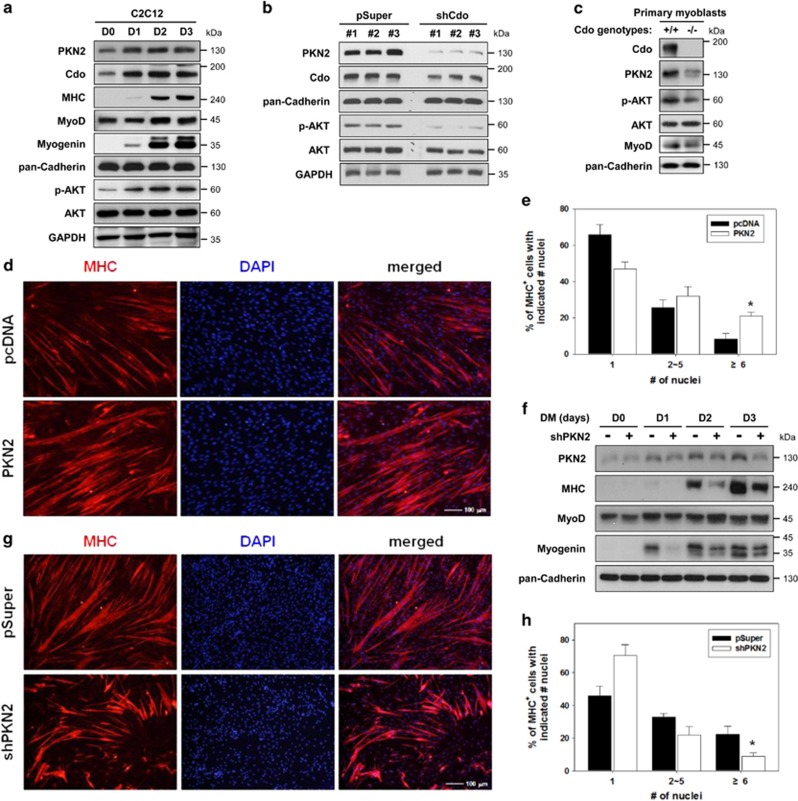
PKN2 levels are elevated during myoblast differentiation and decreased in Cdo-depleted cells with a concurrent reduction in AKT activation. (**a**) C2C12 cells were cultured to near confluency (D0) and induced to differentiate in differentiation medium (DM) for total 3 days (D3). Lysates were immunoblotted with antibodies to PKN2, Cdo, MHC, MyoD, myogenin, phosphorylated-AKT (p-AKT) and AKT. GAPDH and pan-Cadherin serve as loading controls. (**b**) Lysates of C2C12 cells transiently transfected with Cdo or control expression vectors as indicated were immunoblotted with antibodies to PKN2, Cdo, p-AKT and AKT. GAPDH and pan-Cadherin serve as loading controls. (**c**) Immunoblot analysis for the expression of PKN2, p-AKT and AKT proteins in Cdo^+/+^ and Cdo^−/−^ primary myoblasts from hindlimb muscles, and pan-Cadherin serves as a loading control. (**d**) Photomicrographs of C2C12 cells that stably express PKN2 or control vectors were cultured in DM for 2 days, fixed, and immunostained with an antibody to MHC followed by DAPI staining to visualize nuclei. Size bar, 100 *μ*m. (**e**) Quantification of myotube formation shown in (**d**). Values represent means of triplicate determinations ±1 S.D. The experiment was repeated three times with similar results. Significant difference from control, *P<0.01. (**f**) C2C12 cells stably transfected with shPKN2 or control (pSuper) vectors, and cultured to confluency and induced to differentiate for total 3 days. Cell lysates were immunoblotted using antibodies to PKN2, MHC, MyoD, Myogenin and pan-Cadherin as a loading control. (**g**) Photomicrographs of C2C12 cells stably transfected with PKN2 shRNA or control vectors were cultured in DM for 3 days, fixed and immunostained with an antibody to MHC followed by DAPI staining to visualize nuclei. Size bar, 100 *μ*m. (**h**) Quantification of myotube formation by cell lines shown in (**g**). Values represent means of triplicate determinations ±1 S.D. The experiment was repeated three times with similar results. Significant difference from control, *P<0.01

To investigate the function of PKN2 in skeletal myogenesis, C2C12 cells were grown to near-confluency (D0) and induced to differentiate for 3 days (D3), followed by immunoblotting. PKN2 and Cdo proteins were upregulated upon induction of myoblast differentiation that was concurrent with Myogenin induction and stayed high until D3 (Figure 1a). Similarly to PKN2 and Cdo induction, the levels of active phosphorylated AKT (p-AKT) increased, while total AKT levels stayed constant during differentiation (Figure 1a). These data suggest a positive role of PKN2 in AKT activation and myoblast differentiation. To examine the relationship between PKN2 and Cdo, C2C12 cells stably expressing the control (pSuper) or Cdo shRNA expression vectors were analyzed by immunoblotting. Intriguingly, Cdo-depleted cells displayed a stark reduction in PKN2 and p-AKT levels (Figure 1b). In addition, *Cdo*^+/+^ and *Cdo*^−/−^ myoblasts isolated from mouse hindlimbs were induced to differentiate for 2 days by removal of basic fibroblast growth factor (bFGF) and assessed by immunoblotting (Figure 1c). In agreement with the Cdo knockdown result, the levels of PKN2 and p-AKT were decreased substantially without alterations in total AKT levels.

Previously, we have shown that the expression of Cdo and MyoD is dependent on the integrin-mediated cell adhesion. When C2C12 cells were cultured in suspension or on poly-l-lysine (PLL) integrin-independent substratum, the expression of Cdo and downstream signaling pathways, including activation of Cdc42, p38MAPK and AKT were starkly inhibited, resulting in a block of myoblast differentiation.^33^ Thus, we determined whether PKN2 expression was affected in C2C12 cells cultured on normal culture plate or PLL-coated petri-dish and serum-deprived for 36 h. C2C12 cells cultured on PLL exhibited greatly reduced Cdo levels which correlated well with diminished PKN2 and p-AKT expression. In addition, total AKT levels decreased substantially, compared with control cells (Supplementary Figure 1A). The quantification of PKN2 levels and the ratio of p-AKT/AKT from multiple experiments showed that PKN2 expression was reduced roughly to 30% and the ratio of p-AKT/AKT decreased to ~20% of the control levels (Supplementary Figure 1B). The decreased AKT activation in cells on PLL accompanied by a strong accumulation of cleaved PARP with the concurrent reduction in full-length PARP proteins, compared with control cells (Supplementary Figure 1C). Furthermore, cells on PLL showed increased nuclear fragmentation with ~15% of total cells, while only small fraction of control cells exhibited it (Supplementary Figure 1D and E). These data suggest a positive role of PKN2 for AKT activation in cell adhesion-mediated myoblast survival and differentiation.

### Overexpression of PKN2 accelerates myoblast differentiation

In the quantitative RT-PCR analysis with various adult tissues, PKN2 transcripts were detected highly in skeletal muscle, liver, heart and stomach, and modestly in brain and spleen, while it was very low in pancreas (Supplementary Figure 2A). Furthermore, PKN2 was expressed in hindlimb muscles throughout the examined stages with a modest increase until postnatal day 14 (P14) and normalized at P30, which may reflect the fast muscle growth during the first 2 weeks of postnatal life (Supplementary Figure 2B). To examine the function of PKN2 in myogenesis, control or PKN2-overexpressing C2C12 cells were induced to differentiate for 3 days. PKN2-transfected cells generally showed about 3–4-fold increase in PKN2 levels, compared with control cells (Supplementary Figure 2C). PKN2 overexpression accelerated and enhanced the expression of muscle-specific genes, such as Myosin Heavy Chain (MHC), MyoD and Myogenin, relative to that of control cells (Supplementary Figure 2C). Control C2C12/pcDNA3.1 and C2C12/PKN2 cells at D2 were subjected to immunostaining with anti-MHC antibodies to evaluate myotube formation (Figure 1d). The extent of myotube formation was quantified and MHC-positive cells were scored as mononucleate, containing two to five nuclei, or containing six or more nuclei. (Figure 1e). C2C12/pcDNA3.1 cells were ~66% mononucleate, ~25% with two to five nuclei and ~9% with more than six nuclei per myotube (Figures 1d and e). In contrast, C2C12/PKN2 cells exhibited a decrease in the proportion of mononucleate cells and a significant increase in larger myotubes with more nuclei from ~9 to ~21%, compared with that of the control cells (Figures 1d and e). However, the FACS analysis showed no significant effect of PKN2 overexpression on cell death in proliferating or differentiation C2C12 cells (Supplementary Figure 3A). These data suggest that PKN2 promotes myoblast differentiation at morphological as well as biochemical levels.

### PKN2 depletion reduced myoblast differentiation

Next we investigated whether PKN2 induction is a rate-limiting step for myoblast differentiation by PKN2 knockdown. Initially, four different PKN2 shRNA constructs were tested and two of them (#2 and #3) showed reproducible knockdown effects in C2C12 myoblasts which were used interchangeably for further studies (Supplementary Figure 4). C2C12 cells were stably transfected with the control shRNA (pSuper) or shPKN2 expression vector and induced to differentiate for 3 days, followed by immunoblot analysis and immunostaining with anti-MHC antibodies. PKN2 induction upon myoblast differentiation was diminished in C2C12/shPKN2 cells, compared with C2C12/pSuper cells (Figure 1f). PKN2 depletion resulted in delayed myoblast differentiation, evident by reduced expression of MHC and myogenin, compared with C2C12/pSuper cells (Figure 1f). In addition, the MHC immunostaining of these cells revealed that PKN2 induction is critical for myotube formation (Figure 1g). C2C12/shPKN2 cells formed smaller myotubes with fewer nuclei per myotube, compared with C2C12/pSuper cells. C2C12/shPKN2 cells were 70% mononucleate, ~20% with two to five nuclei and ~10% with more than six nuclei per myotube, while C2C12/pSuper cells were 46% mononucleate, 32% small myotubes with two to five nuclei, 22% with more than six nuclei (Figure 1h). This effect of PKN2 depletion on myoblast differentiation appears to be independent of cell death. There was no obvious effect on cell death index (Supplementary Figure 3B). Interestingly, unlike the effect of Cdo depletion on PNK2 expression (Figure 1c), PKN2 depletion did not affect Cdo protein levels (Supplementary Figure 5). These results indicate that PKN2 is required for efficient myoblast differentiation.

### PKN2 interacted with Cdo through its C-terminal region

**Figure 2:**
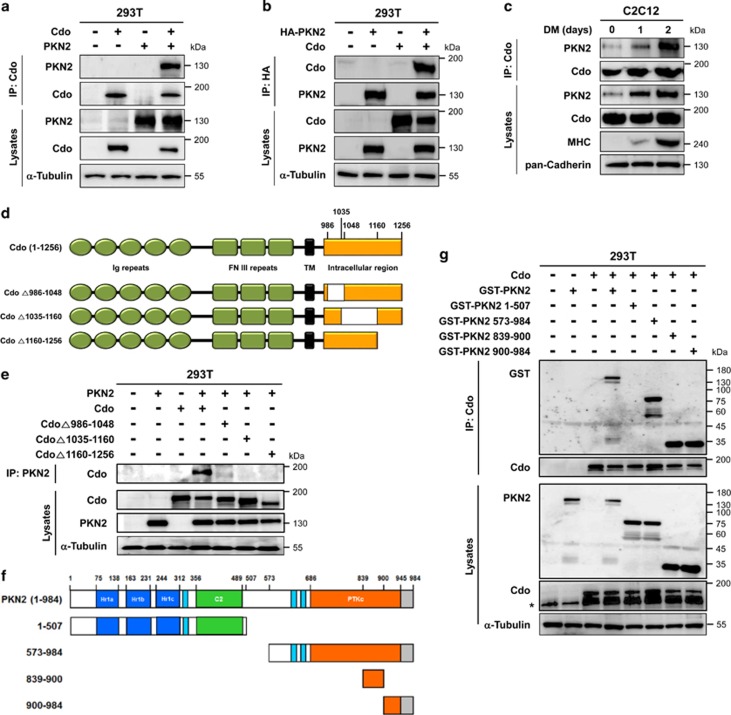
PKN2 forms complexes with Cdo. (**a**) Lysates of 293T cells transiently transfected with Cdo, PKN2 or control expression vectors as indicated were immunoprecipitated with an antibody to Cdo. The precipitates and total lysates were immunoblotted with antibodies against Cdo and PKN2, and to *α*-tubulin as a loading control. (**b**) Lysates of 293T cells transiently transfected with Cdo, HA-PKN2 or control expression vectors as indicated were immunoprecipitated with an antibody to HA. The precipitates were immunoblotted with antibodies against Cdo or HA. Total lysates were immunoblotted with antibodies to Cdo and PKN2, and to *α*-tubulin as a loading control. (**c**) Lysates of C2C12 cells that were proliferating in growth medium (D0) or in DM for the indicated time were immunoprecipitated with antibodies to Cdo and immunoblotted with antibodies to PKN2 or Cdo. Total lysates were also immunoblotted with antibodies to PKN2, Cdo and MHC, and to pan-Cadherin as a loading control. (**d**) Schematic representation of rat Cdo, the amino acid numbers indicating the deletion constructs. (**e**) Lysates of 293T cells transiently transfected with Cdo, Cdo deletion mutants, PKN2 or control expression vectors as indicated were immunoprecipitated with PKN2 antibodies and then immunoblotted with antibodies to Cdo. Total lysates were also immunoblotted with antibodies to Cdo or PKN2, and to *α*-tubulin as a loading control. (**f**) The schematic representation depicts the domain structure of PKN2 and the deletion of the specific domain. (**g**) Lysates of 293T cells transiently transfected with GST-tagged PKN2, its deletion mutants, Cdo or control expression vectors as indicated were immunoprecipitated with Cdo antibodies and then immunoblotted with antibodies to Cdo or GST. Total lysates were also immunoblotted with antibodies to Cdo or PKN2, and to *α*-tubulin as a loading control. Asterisk marks a nonspecific band in the Cdo blot

The fact of the concomitant expression of PKN2 and Cdo and the partial dependency of PKN2 expression on Cdo, we investigated a potential interaction between Cdo and PKN2. 293T cells transfected with Cdo and PKN2 were subjected to co-immunoprecipitation analysis. PKN2 and Cdo co-immunoprecipitated reciprocally when coexpressed in 293T cells (Figures 2a and b). Furthermore, endogenous PKN2 proteins were co-immunoprecipitated with Cdo in C2C12 cells during differentiation however the interaction of PKN2 with Cdo was enhanced greatly and exponentially in differentiating myoblasts, correlating with increasing PKN2 expression (Figure 2c), suggesting that Cdo and PKN2 interact physically in differentiating myoblasts.

Next, we determined the structural requirement mediating Cdo interaction with PKN2. 293T cells were transfected with PKN2 and the full-length Cdo or intracellular deletion constructs (CdoΔ986-1048, CdoΔ1035-1160 and CdoΔ1160-1256), as indicated in Figure 2d, followed by immunoprecipitation. The full-length Cdo was co-immunoprecipitated well with PKN2, while all three Cdo mutants failed to do so, suggesting that the intracellular region of Cdo is essential for PKN2 binding (Figure 2e). Previously, we have shown that the intracellular region of Cdo is required for myoblast differentiation and the intracellular deletion mutants behaved similarly to a loss-of-function mutant in myoblast differentiation.^9^ This supports that the interaction of Cdo with PKN2 might be required for Cdo-mediated myoblast differentiation.

To identify the domain of PKN2 responsible for Cdo interaction, we have constructed four different GST-tagged expression vectors harboring following PKN2 regions (Figure 2f): (1) amino acid (AA) 1-507 containing the N-terminal region and C2-like domain; (2) AA573-984 harboring the catalytic core and the C-terminal region; (3) AA839-984 containing the middle of catalytic core and the C-terminal region; and (4) AA900-984 containing the C-terminal region. These GST-tagged constructs were co-transfected with Cdo into 293T cells and lysates were subjected to immunoprecipitation with anti-Cdo antibodies, followed by immunoblot analysis. GST-tagged full-length and AA573-984, AA839-984 and AA900-984 of PKN2 proteins were immunoprecipitated robustly with anti-Cdo antibodies (Figure 2g). In contrast, GST-PKN2/1-507 failed to interact with Cdo (Figure 2g), suggesting that the C-terminal region comprised of AA839-984 of PKN2 mediates the interaction with Cdo.

### The C-terminal region of PKN2 was sufficient to enhance myoblast differentiation

**Figure 3:**
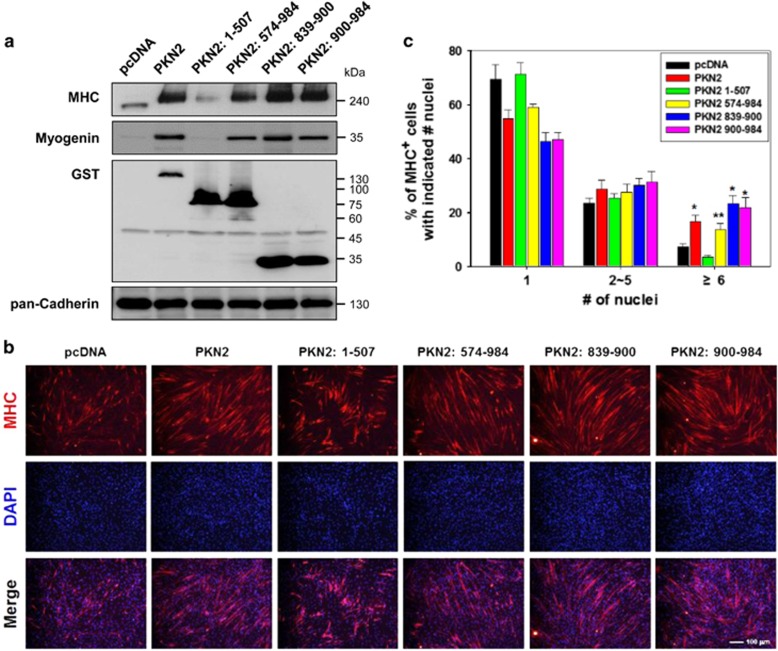
The C-terminal region of PKN2 is sufficient to enhance myoblast differentiation. (**a**) C2C12 cells were stably transfected with GST-tagged PKN2 and its deletion mutants, or control expression vectors (pcDNA). Lysates of these cell lines were immunoblotted with antibodies to GST to reveal the level of ectopic expression of PKN2 and PKN2 mutants. In addition, lysates were immunoblotted with antibodies to MHC, Myogenin and pan-Cadherin as a loading control. (**b**) C2C12 cells shown in **a** were cultured in DM for 2 days, fixed and immunostained with an antibody to MHC followed by DAPI staining to visualize nuclei. Size bar, 100 *μ*m. (**c**) Quantification of myotube formation by cell lines shown in (**b**). Values represent means of triplicate determinations ±1 S.D. The experiment was repeated three times with similar results. Significant difference from control, *P<0.01, **P<0.05

To explore the functional significance of PKN2 interaction with Cdo, stable C2C12 cells expressing the full length or domains of PKN2 were induced to differentiate for 2 days. C2C12 cells expressing the full-length PKN2, PKN2/574-984, PKN2/839-900 or PKN2/900-984 displayed greatly enhanced MHC and Myogenin expression (Figure 3a). Considering the relatively lower expression of the full-length PKN2, the full-length PKN2 appeared to be most effective to induce MHC and myogenin expression. In contrast, PKN2/1-507 protein deficient for Cdo interaction slightly decreased MHC and myogenin expression (Figure 3a). Similar results were observed in myotube formation (Figures 3b and c). The full-length PKN2-overexpressing cells formed proportionally more of larger myotubes containing six or more nuclei, compared with control cells. Cells expressing PKN2/839-900 and PKN2/900-984 formed slightly but significantly more of larger myotubes containing six or more nuclei (23.3% and 21.8%, respectively), compared with the full-length PKN2 expressing cells (16.7%) (Figure 3c). Consistently with MHC expression, PKN2/1-507 had no effect on myotube formation (Figures 3b and c). These data suggest that the C-terminal region of PKN2 is sufficient to induce myoblast differentiation and this promyogenic function of PKN2 appears to be independent of its kinase activity.

### The C-terminal region of PKN2 was sufficient for AKT activation in myoblast differentiation

**Figure 4:**
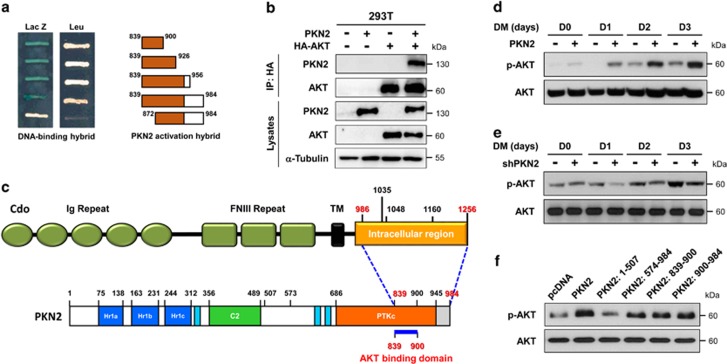
The C-terminal region of PKN2 is sufficient for the AKT activation. (**a**) Yeast two-hybrid analysis. The DNA-binding hybrid containing the full-length AKT and the activation hybrids encoding the indicated regions of PKN2 C-terminus were utilized for the interaction ability. The growth on the media lacking leucine and the blue staining for *β*-galactosidase activity are indicative of the interaction between AKT and PKN2 fragments. Note that the PKN2 lacking AA839-872 failed to interact with AKT. (**b**) Lysates of 293T cells transfected with PKN2, HA-AKT or control vector were subjected to immunoprecipitation with HA and immunoblotting with PKN2 or HA antibodies. Total lysates were also immunoblotted with antibodies to PKN2 and AKT, and to *α*-tubulin as a loading control. (**c**) Schematic diagram of the interacting domains with Cdo and PKN2. (**d**) C2C12 cells were stably transfected with PKN2 and its deletion mutants or control expression vectors (pcDNA). Lysates of these cell lines were immunoblotted with antibodies to p-AKT and AKT. (**e**) C2C12 cells were stably transfected with PKN2 overexpression and control vector (pBp), and cultured to confluency and induced to differentiate for total 3 days. Cell lysates were immunoblotted with antibodies to p-AKT and AKT. (**f**) C2C12 cells stably expressing shPKN2 or control expression vectors (pSuper) were cultured to confluency and induced to differentiate for total 3 days. Cell lysates were immunoblotted with antibodies to p-AKT and AKT

Next we assessed the interaction between PKN2 and AKT in myoblast differentiation. In agreement with the previous report,^31^ a yeast two-hybrid screening suggested that the minimal region between AA839 to 900 of PKN2 interacted with AKT (Figure 4a). PKN2 and AKT formed a complex when coexpressed in 293T cells (Figure 4b). In a previous report, the cleaved form of PKN2 containing the C-terminal region of AA862-908 by Caspases interacted with and inhibited AKT in the induction of apoptosis.^31^ AKT activation and cell survival are essential for efficient myoblast differentiation.^7, 34^ The current data suggest that the C-terminal region of PKN2 mediates interaction with Cdo and AKT (Figure 4c). To determine the effect of PKN2 overexpression or knockdown on AKT activation, similar experimental sets shown in Figure 3 were analyzed for AKT activation. In contrast to the reported inhibitory role of PKN2 for AKT in response to drug-induced apoptosis, the full-length PKN2 elevated p-AKT levels during myoblast differentiation (Figure 4d). Conversely, PKN2-depletion diminished AKT activation during myoblast differentiation (Figure 4e). Furthermore, control or C2C12 cells expressing the full-length PKN2 or PKN2 domains were induced to differentiate for 2 days and analyzed for AKT activation (Figure 4f). The levels of p-AKT correlated well with the differentiation efficiency of these cells (Figures 3a and c). PKN2/1-507 failed to activate AKT which correlated with the failure to enhance differentiation. However, the expression of other PKN2 domains strongly enhanced AKT activation and myotube formation. These data suggest that PKN2 activates AKT during myoblast differentiation and the interaction of PKN2 with Cdo and AKT is critical for AKT activation and the promyogenic effect.

### PKN2, Cdo and APPL1 augmented cooperatively activities of AKT and the MyoD-responsive reporter

**Figure 5:**
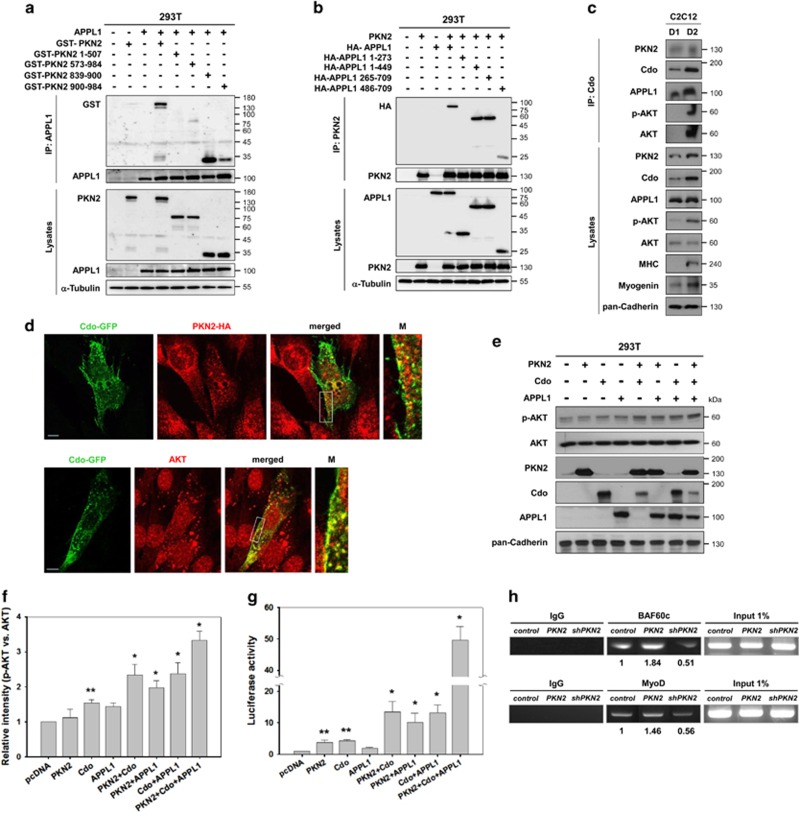
PKN2, Cdo and APPL1 cooperatively activate AKT and the MyoD-responsive reporter activities. (**a**) Lysates of 293T transiently transfected with GST-tagged PKN2, its deletion mutants, APPL1 or control expression vectors as indicated were immunoprecipitated with APPL1 antibody and then immunoblotted with antibodies to GST or APPL1. Total lysates were also immunoblotted with antibodies to PKN2 or APPL1, and to *α*-tubulin as a loading control. (**b**) Lysates of 293T transiently transfected with HA-tagged APPL1, its deletion mutants, PKN2 or control expression vectors as indicated were immunoprecipitated with PKN2 and then immunoblotted with antibodies to HA or PKN2. Total lysates were also immunoblotted with antibodies to APPL1 or PKN2, and to *α*-tubulin as a loading control. (**c**) Lysates of C2C12 cells at D1 and D2 were immunoprecipitated with a Cdo antibody and immunoprecipitates and total cell lysates were assessed by immunoblotting with indicated antibodies. Cadherin expression serves as a loading control. (**d**) C2C12 cells transfected with Cdo-GFP alone or with HA-PKN2 expression vectors were induced to differentiate for one day and subjected to immunostaining with antibodies to HA (upper panel) or AKT (lower panel). The enlarged images of the boxed areas are shown in the right panels. Size bar, 10 *μ*M. (**e**) 293T cells were transiently transfected with expression vectors for PKN2, Cdo, APPL1 or combination of these vectors as indicated. The lysates were immunoblotted with antibodies to p-AKT, total AKT, PKN2, Cdo, APPL1 and pan-Cadherin as a loading control. (**f**) Quantification of three blots similar immunoblots to those shown in (**e**). The intensity of p-AKT was quantified with the values obtained from control vector-transfected cells set to 1.0. The values represent the means of triplicate determinations±1 S.D. The experiment was repeated three times with similar results. Significant difference from control, *P<0.01, **P<0.05. (**g**) 10T1/2 cells were co-transfected with a MyoD-responsive luciferase reporter and the expression vectors for MyoD and *β*-galactosidase as an internal control. In addition, control, PKN2, Cdo and/or APPL1 expression vectors were co-transfected as indicated for 24 h later, the reporter activities were measured and normalized relative to the internal control. The experiment was performed as triplicates and repeated three times with similar results. *P<0.01, **P<0.05. (**h**) Chromatin immunoprecipitation with anti-MyoD or anti-BAF60c antibodies was performed with C2C12 cells transfected with pSuper, PKN2 or shPKN2. ChIP DNA was assessed by quantitative PCR with primers that specially recognize the MyoD-responsive elements in the Myogenin promoter. All ChIP analysis were performed with three independent chromatin preparations

In our previous study, APPL1 was identified as an AKT interacting protein in yeast two-hybrid screening and shown to promote AKT activation and myoblast differentiation through interaction with Cdo.^7^ In the same screening, PKN2 was also identified as an AKT interacting protein. Thus we investigated whether PKN2 physically interacts with APPL1. 293T cells were co-transfected with APPL1 and GST-tagged PKN2 constructs as shown in Figure 4 or GST-PKN2 with HA-tagged full-length or various regions of APPL1.^7^ The full-length PKN2 was coprecipitated with APPL1 (Figure 5a) or APPL1 was coprecipitated with GST-tagged full-length PKN2 (Figure 5b). However GST-PKN2/1-507 was not coprecipitated with APPL1 while other PKN2 constructs showed variable interaction efficiency. GST-PKN2/573-984 precipitated weakly with APPL1, while GST-PKN2/839-900 proteins precipitated with APPL1 more than the full-length or other regions of PKN2, suggesting that the region between AA839 to AA900 of PKN2 mediates a strong interaction with APPL1 (Figure 5a). In a converse experiment, HA-APPL1/1-273 failed to interact with PKN2, while the full-length and other region-containing APPL1 proteins precipitated with GST-PKN2. However APPL1/486-709 exhibited a weak interaction with GST-PKN2, suggesting that the region of AA273-449 is the minimal region responsible for PKN2 interaction (Figure 5b). These data suggest that APPL1 and PKN2 can form complexes.

We next examined whether endogenous Cdo, PKN2, APPL1 and AKT proteins form complexes in differentiating C2C12 cells by immunoprecipitation with Cdo antibodies. PKN2 and APPL1 were coprecipitated with Cdo in C2C12 cells both at D1 and D2; however, more proteins were coprecipitated in C2C12 cells at D2 coinciding with induction of MHC expression and high myogenin levels (Figure 5c). In addition, AKT and p-AKT proteins were readily detected in precipitates at D2. To examine whether Cdo colocalizes with PKN2 and AKT, C2C12 cells were transfected with Cdo-GFP alone or together with PKN2-HA. 24 h later, cells were switched into differentiation medium for one day and subjected to immunostaining with HA or AKT antibodies, followed by confocal microscopy. Cdo-GFP was detected in the cytoplasm and along the plasma membrane as a punctuated pattern. A fraction of PKN2 and AKT proteins colocalized with Cdo-GFP mainly in the vicinity of the plasma membrane (Figure 5d). These data suggest that Cdo, PKN2 and AKT form complexes in differentiating myoblasts.

To further define, Cdo, PKN2 and APPL1 were co-transfected in 293T cells as indicated and analyzed the expression levels of transfected proteins, p-AKT and total AKT (Figure 5e). The relative intensities of p-AKT *versus* total AKT signals were quantified and plotted in Figure 5f. In agreement with our published work,^7^ the expression of Cdo enhanced slightly but significantly p-AKT levels. However, 293T cells expressing PKN2 or APPL1 singly did not exhibit any significant AKT activation. In contrast, the expression of Cdo together with either PKN2 or APPL1 resulted in roughly 2.4-fold increase in p-AKT levels, relative to control cells, while about 1.9-fold increase in p-AKT levels was observed in cells coexpressing PKN2 and APPL1 (Figure 5f). 293T cells expressing all three proteins exhibited the strongest p-AKT levels with ~3.4-fold increase, compared with control cells.

To further define the function of these proteins, 10T1/2 cells were co-transfected with a MyoD-responsive luciferase reporter (4RTK-luc) in combination of constructs as indicated and 24 h later, cells were switched into the differentiation medium for one day, followed by luciferase assay. The expression of PKN2, Cdo or APPL1 singly enhanced modestly MyoD-reporter activities, which were greatly elevated by cotransfection of these proteins in a double combination (Figure 5g). Interestingly, the coexpression of all three proteins enhanced massively MyoD-reporter activities, suggesting that Cdo, PKN2 and APPL1 function cooperatively in MyoD activation. Furthermore, control C2C12, C2C12/PKN2 or C2C12/shPKN2 cells were induced to differentiate for 1 day and subjected to chromatin immunoprecipitation assay for the enrichment of BAF60c and MyoD in the myogenin promoter containing MyoD-responsive sequences (CANNTG, called E-boxes). PKN2 overexpression enhanced the recruitment of BAF60c and MyoD to the promoter region of the myogenin gene (Figure 5h). In contrast, PKN2 depletion decreased the enrichment of BAF60c and MyoD to the myogenin promoter. Taken together, the data shown in this study suggest that PKN2 promotes myoblast differentiation through interaction with Cdo and AKT leading to AKT activation and MyoD-mediated gene expression.

## Discussion

Cell proliferation and differentiation are mutually exclusive processes that are regulated by distinct extracellular cues triggering activation of often identical intracellular signaling pathways, such as AKT signaling.^34, 35^ AKT signaling is involved in regulation of both proliferation- as well as differentiation-related cell survival.^18, 36, 37^ In connection with this, AKT hyperactivation is implicated in aberrant cell proliferation and growth.^38^ Thus, it is likely that distinct mechanisms for AKT activation might exist under the specific cellular contexts. Our data in this study demonstrate that PKN2 together with APPL1 might be specifically involved in the differentiation-specific activation of AKT and Cdo-mediated myoblast differentiation. In agreement with this notion, PKN2 expression was enhanced during myoblast differentiation (Figure 2c) and PKN2 overexpression did not show enhanced AKT activation in proliferating C2C12 and 293T cells (data not shown). Furthermore, the effect of PKN2 on AKT activation seemed to be dependent on the presence of Cdo expression. Consistently, 293T cells, which have no detectable levels of Cdo expression showed no effect on AKT activation when PKN2 was overexpressed (Figure 5e). In contrast, coexpression of Cdo and PKN2 in 293T cells resulted in significantly elevated AKT activation. The detailed mode of AKT activation mediated by Cdo is currently unknown. However the complex formation of Cdo and PKN2 appeared to be a critical event to activate AKT and myogenic differentiation.

Although we did not observe any effect on cell death of Cdo-depleted C2C12 cells under growing or differentiation conditions, myoblasts cultured on PLL under serum deprivation for 36 h revealed the increases in cleaved PARP and nuclear fragmentation, and also exhibited greatly reduced levels of Cdo, PKN2 and p-AKT. These results are contradictory with the previously proposed role of PKN2 in apoptosis induced by Fas ligation, anti-fungal or anti-cancer drugs.^39^ It has been reported that PKN2 is cleaved at AA700 by caspases into the C-terminal fragment comprising AA700-984 which interacts with and inhibits AKT.^31^ Since we failed to observe any apoptotic effects when the full-length PKN2 or the C-terminal fragments of PKN2 were overexpressed in C2C12 cells, this apoptotic effect of PKN2 might be cell type- or cellular context-dependent.

In the present study, we identified Cdo and APPL1 as new PKN2-binding proteins. The interaction of PKN2 with Cdo and APPL1 appeared to be mediated through its C-terminal region (AA839-984 and AA839-900, respectively). According to the published work, this region has been shown to contain a domain, which is responsible for AKT interaction (AA862-900).^31^ These short C-terminal PKN2 fragments appeared to be devoid of the full-length kinase domain, suggesting that PKN2 might function as a scaffold protein. Previously, it has been reported that the C-terminal 77 amino acids of PKN2 interact with the upstream kinase PDK1, resulting in activation of PKN2.^40^ Since the PKN2/AA839-900 fragment which does not contain the binding region to PDK1^41^ enhanced AKT activation and myoblast differentiation, PDK1 appears not to be directly involved in the promyogenic function of PKN2 or AKT activation during myogenic differentiation. The N-terminal region of PKN2 interacts with Rho or Rac GTPases and functions as a downstream effector to regulate cell migration.^28^ Our data demonstrate that the N-terminal region of PKN2 that does not interact with Cdo, AKT or APPL1 appears to be dispensable for Cdo-mediated AKT activation and myogenesis. In addition, this N-terminal domain of PKN2 possesses inhibitory activities for PKN2 kinase activity and PDK1 interaction.^40^ Since the ectopic expression of the N-terminal region of PKN2 did not show obvious dominant-negative effects on the differentiation-associated AKT activation and myogenesis, Cdo/PKN2 complexes might function independently of PKN2 kinase activities and PDK1. Our study suggests an essential role of PKN2 as a positive mediator for the regulation of AKT activity during myoblast differentiation. The intrinsic control of AKT activity through the complex association of PKN2 with Cdo and APPL1 would provide insights into the functional regulation of cell adhesion signaling pathways in skeletal muscle differentiation.

## Materials and Methods

### Reagents

Fetal bovine serum (FBS) and Dulbecco modified Eagle's medium (DMEM) were purchased from Thermo Scientific (Waltham, MA, USA). Horse serum (HS) are obtained from WelGene (Daegu, Korea). Lipofectamin 2000 was obtained from Invitrogen (Carlsbad, CA, USA). Antibodies used in this study are as following: p-AKT (4060), AKT (9272), PARP (9542), cleaved PARP (5625) (Cell Signaling Technology, Boston, MA, USA), PKN2 (sc-27197), MyoD (sc-32758), myogenin (sc-576), APPL1 (sc-67402), GST (sc-138), HA (sc-7392), GAPDH (sc-137179), *α*-tubulin (sc-5286) (Santa Cruz Biotechnology, Santa Cruz, CA, USA), Cdo (AF2429; R&D systems), MHC (MF-20; Developmental Studies Hybridoma Bank) and pan-Cadherin (C3678; Sigma-Aldrich, St Louis, MO, USA). For chromatin immunoprecipitation assays, ChiP-grade MyoD and BAF60c antibodies (Santa Cruz Biotechnology) are used. The shRNAs of PKN2, poly-l-lysine and all other chemicals were obtained from Sigma-Aldrich.

### Cell culture and expression vectors

Myoblast C2C12 cells, primary myoblasts, embryonic fibroblast 10T1/2 cells and embryonic kidney 293T cells were cultured as described previously.^7^ To induce differentiation of C2C12 myoblasts, cells at near confluence were changed from DMEM containing 15% FBS (growth medium, GM) to DMEM containing 2% HS (differentiation medium, DM), and myotube formation was observed at 2 or 3 days of differentiation. The efficacy of myotube formation was quantified by a transient differentiation assay as previously described.^1^ To generate C2C12 cells that stably overexpress Cdo, APPL1, PKN2, mutant forms of PKN2, or shRNAs against PKN2 or Cdo, cells were transfected with the indicated expression vectors and Lipofectamine 2000, and cultures were selected in puromycin-containing medium. Four different PKN2 shRNAs were screened for their effectiveness by transfection into 293T cells and #2 and #3 have shown the most reproducible knockdown effect. The sequences are as following: #1: 5′-CCGGGTCCACGTCAAAGTATGATATCTCGAGATATCATACTTTGACGTGGACTTTTTG-3′, #2: 5′-CCGGTACTTTGGAAGTTCGTCTTATCTCGAGATAAGACGAACTTCCAGTATTTTTG-3′, #3: 5′-CCGGGCAGGAATTAAATGCACATATCTCGAGATATGTGCATTTAATTCCTGCTTTTT-3′, #4: 5′-CCGGGCACATTCATACTGATGTCTTCTCGAGAAGACATCAGTATGAATGTGCTTTTT-3′.

For the PKN2 mutant study, the human *PKN2* gene was amplified by reverse transcription polymerase chain reaction (RT-PCR) of mRNAs purified from human embryonic kidney fibroblast cells. Full-length (AA1-984) and mutant forms of *PKN2* (AA1-507, AA573-984, AA839-900 and AA900-984) were inserted into mammalian expression vector pcDNA-GST. *Cdo* deletion mutants (CdoΔ986-1048, CdoΔ1035-1160 and CdoΔ1160-1256), *Cdo* intracellular constructs and the sequence for the pSuper/*Cdo*-shRNA construct were described previously.^1^ Full-length *APPL1* construct and *APPL1* mutants (AA1-499, AA1-273, AA265-709 and AA486-709) were described previously.^7^

### Western blot analysis and immunoprecipitation

Western blot analyses were carried out as previously described.^1^ Briefly, cells were lysed in cell lysis buffer (10 mM Tris-HCl, pH 7.2, 150 mM NaCl, 1 mM EDTA, 1% Triton X-100) containing complete proteinase inhibitor cocktail (Roche, Basel, Germany) and SDS-polyacrylamide gel electrophoresis (SDS-PAGE) was performed. For immunoprecipitation assay, 293T cells were transfected with combination of Cdo and HA-tagged PKN2. Thirty-six hours after transfection, whole cell extracts were incubated with anti-HA and protein G agarose beads (Roche Diagnostics) overnight at 4 °C. The beads were washed three times with extraction buffer and resuspended in extraction buffer, and samples were analyzed by western blotting.

### Immunocytochemistry and microscopy

Immunostaining for MHC expression was performed as described previously.^1^ Briefly, C2C12 cells were transfected with pcDNA, pSuper, PKN2, PKN2 deletion mutants or shPKN2, fixed with 4% paraformaldehyde for 20 min, permeabilized with 0.5% Triton X-100 in phosphate-buffered saline (PBS), blocked, and stained with anti-MHC, followed by an Alexa Fluor 568-conjugatd secondary antibody (Invitrogen). Images were captured and processed with a Nikon ECLIPSE TE-2000U microscope and NIS-Elements F software (Nikon, Tokyo, Japan). Quantitative differentiation assay was performed for at least three independent experiments. For colocalization studies, C2C12 cells transfected with Cdo-GFP or PKN2-HA expression vectors were cultured on collagen-coated cover glass (Marienfeld superior) for 2 days, followed by immunostaining as described above. Primary antibodies used are anti-HA (1:500, AbFrontier, Seoul, South Korea) and anti-Akt (1:200, Cell signaling). Confocal images were obtained and analyzed with LSM-710 META confocal microscope system (Carl Zeiss, MicroImaging GmbH, Göttingen, Germany).

### Luciferase assay

10T1/2 cells were seeded in 12-well plates at a density of 4 × 10^4^ cells per well. Twenty-four hours after seeding, cells were transfected using Lipofectamine 2000 with 100 ng of the reporter plasmid of MyoD-Luc and co-transfected with 50 ng of MyoD. Twelve hours later, transfected cells were transferred into GM, harvested, and firefly luciferase activity was determined using a Luminometer with Luciferase Reporter Assay System (Promega, Sunnyvale, CA, USA). Experiments were performed in triplicates and repeated at least three times independently.

### Yeast two-hybrid assay

A cDNA encoding AKT was ligated to the LexA DNA-binding domain in the pEG202 vector, and cDNA fragments encoding the PKN2 C-terminal region were ligated to the B42 activation domain in the pJG4-5 vector. Yeast strain EGY48 (Clontech, Seoul, South Korea) was sequentially transformed with these vectors, and streaked on plate lacking leucine or containing X-gal to monitor the reporter activity.

### Chromatin immunoprecipitation (ChIP) assay

ChIP analysis was performed on C2C12 cells transfected with pSuper, PKN2, or shPKN2 using the EZ-ChIP kit (Upstate Biotechnology, Lake Placid, NY, USA) according to the manufacturer's instructions. Quantitative PCR analysis were performed on immunoprecipitated DNA and normalized to total chromatin input or total histone H3. Primers for the Myogenin promoter region used in this study are following: 5′-GAATCACATGTAATCCACTGGA-3′ and 5′-ACGCCAACTGCTGGGTGCCA-3′. After amplification, PCR products were separated on 1% agarose gels and visualized by ethidium bromide.

### Statistical analysis

The experiments were carried out independently at least three times. The participants' *t*-test was used to access the significance of the difference between two mean values. *P<0.01 and **P<0.05 were considered to be statistically significant.

## Abbreviations

bFGF: basic fibroblast growth factor
Cdo: cell adhesion molecule-related downregulated by oncogene
ChIP: Chromatin immunoprecipitation
DM: differentiation medium
GAPDH: glyceraldehyde 3-phosphate dehydrogenase
GM: growth medium
IGF: insulin-like growth factor
MEF2: myocyte enhancer factor 2
MHC: myosin heavy chain
PKN2: protein kinase C-related kinase 2
PLL: poly-l-lysine
