# Supplementary material for: PKN2 and Cdo interact to activate AKT and promote myoblast differentiation
Source: Cell Death Dis. 2016 Oct 20;7(10):e2431–. doi: 10.1038/cddis.2016.296 (PMC5133968; doi:10.1038/cddis.2016.296)
Supplement: Supplementary Information [file cddis2016296x1.docx]

**Materials and Methods**

**Cell culture**

Hindlimb and satellite cells isolated from Cdo^+/+^ and Cdo^-/-^ mice were cultured as described previously.^1^ Cells were grown in F10 medium containing 20% FBS and bFGF (100 ng/mL) and induced to differentiate without bFGF.

**Annexin V/PI staining assay**

Annexin V/PI double staining was carried out with the Annexin V-FITC Apoptosis Detection kit (BD Biosciences) according to the manufacturer’s protocol, and samples were analyzed using a FACS Calibur flow cytometer (Becton Dickinson) and Cell Quest 2.0 analysis software.

**Supplementary figure legends**

**Supplementary Figure 1** PKN2 levels were decreased in Cdo-depleted cells accompanied by diminished AKT activation. **(A)** Lysates of C2C12 cells were cultured in PLL-coated plates were immunoblotted with antibodies to PKN2, Cdo, p-AKT, AKT and pan-Cadherin as a loading control. **(B)** Quantification of the three immunoblots, one of which was shown in panel A. The relative intensity of PKN2 and p-AKT signals to pan-Cadherin and total AKT signals, respectively, was quantified and the CP value was set to 1.0. Values represent the means of triplicate determinations ± SD. Significant difference from CP group, **p* < 0.01. **(C)** Lysates of C2C12 cells cultured in PLL-coated plates were immunoblotted with antibodies to PARP, cleaved PARP, and GAPDH as a loading control. **(D)** DAPI staining analysis of C2C12 cells cultured in PLL-coated plates. Arrows indicate nuclei of apoptotic cells. **(E)** Quantification of apoptotic cells. Values represent the means of triplicate determinations ± SD. Significant difference from CP group, **p* < 0.01.

**Supplementary Figure 2** Overexpression of PKN2 accelerated myoblast differentiation. **(A)** RT-PCR analysis of the PKN2 expression in different organs of mice. **(B)** RT-PCR analysis of hindlimb muscle from E15.5 embryos and P1, P5, P7, P14 and P30 mice for the expression of PKN2 and 18S rRNA as a loading control. Values calculate the ratio of PKN2 versus 18S rRNA and represent means of triplicate determinations ±1 SD. **(C)** C2C12 cells stably transfected with PKN2 or control (pcDNA-3.0) vectors, and cultured to confluency and induced to differentiate for total three days. Lysates were immunoblotted with antibodies to PKN2, MHC, MyoD and Myogenin, and to pan-Cadherin as a loading control.

**Supplementary Figure 3** Flow cytometry analysis of cell death with Annexin V/PI. **(A)** C2C12 cells were transiently transfected with PKN2 expression or control vectors and cultured in GM and DM. **(B)** C2C12 cells were transiently transfected with shPKN2 or control vectors and cultured in GM and DM.

**Supplementary Figure 4** The expression of PKN2 and MHC proteins in C2C12 cells expressing the control pSuper or PKN2 shRNA (shPKN2) at the differentiation day 2. Note all 4 shRNAs showed knockdown effects on PKN2 proteins which resulted in decreased expression of MHC.

**Supplementary Figure 5** The expression of Cdo in C2C12 cells expressing the control pSuper or PKN2 shRNA at the differentiation day 1.

**Supplementary reference**

1. Cole F, Krauss RS. Microform holoprosencephaly in mice that lack the Ig superfamily member Cdon. *Curr Biol* 2003, **13**(5)**:** 411-415.
