# Supplementary figures and images for: PKN2 and Cdo interact to activate AKT and promote myoblast differentiation
Source: Cell Death Dis. 2016 Oct 20;7(10):e2431–. doi: 10.1038/cddis.2016.296 (PMC5133968; doi:10.1038/cddis.2016.296)

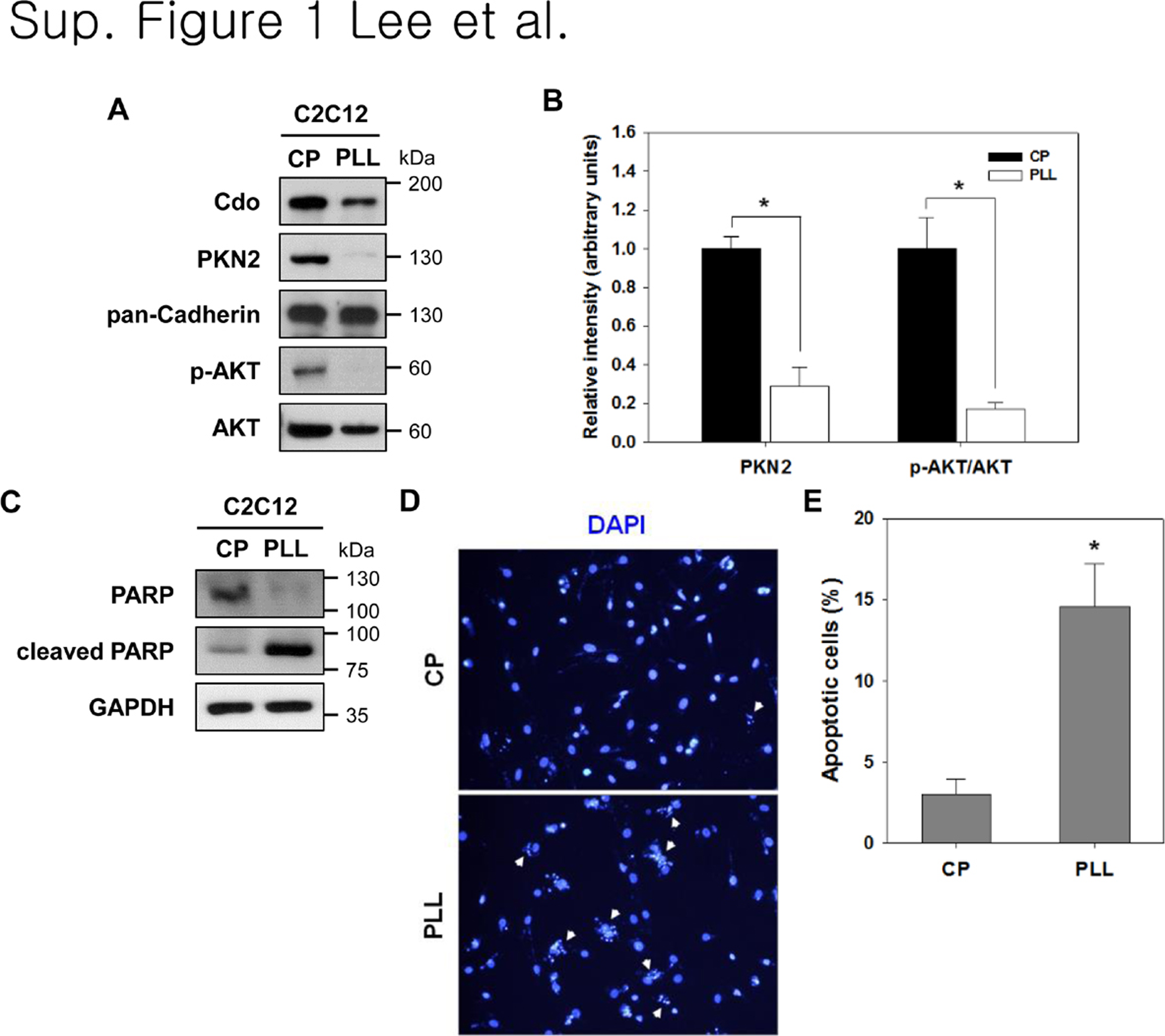

Supplement: Supplementary Figure 1 [file cddis2016296x2.tif]

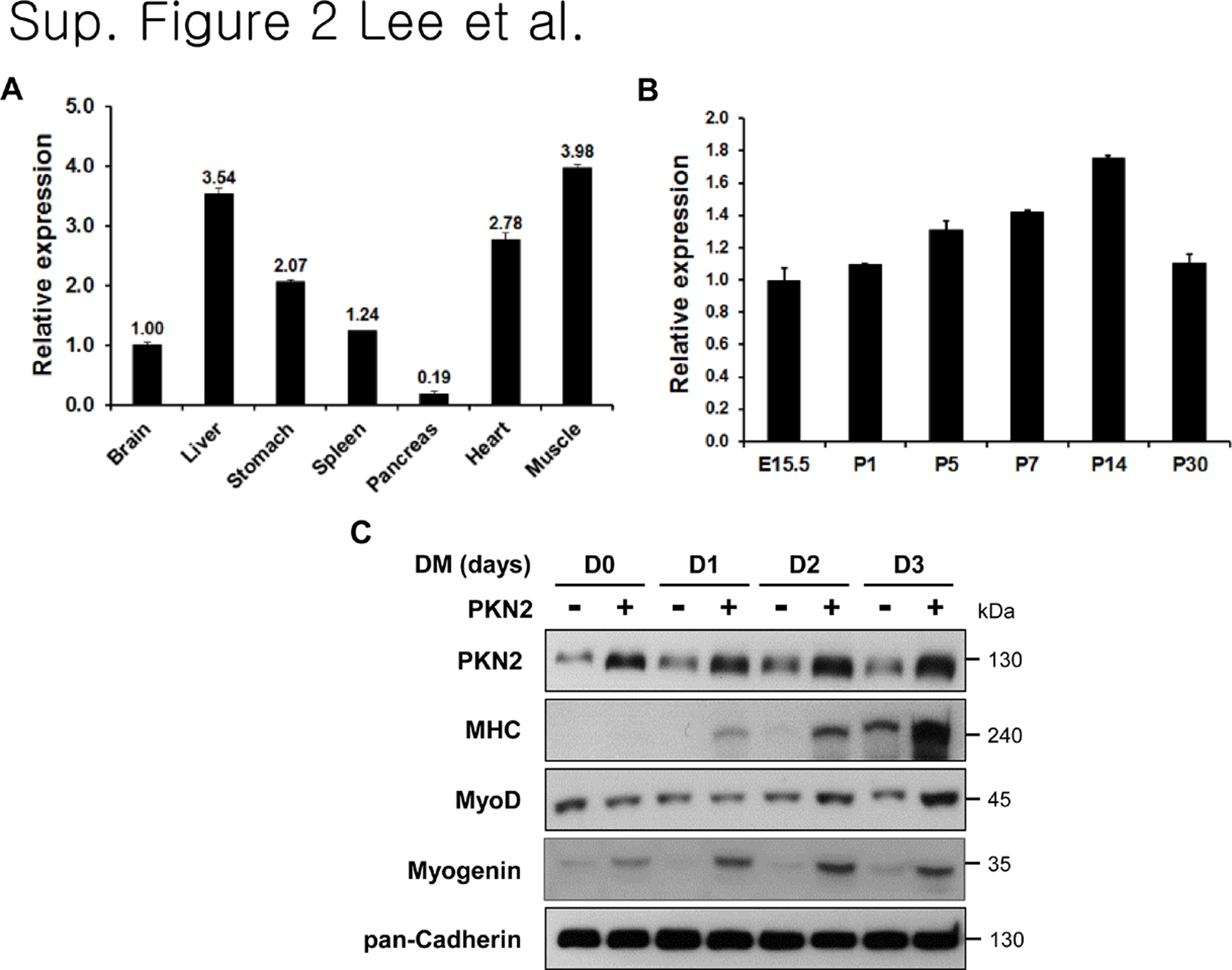

Supplement: Supplementary Figure 2 [file cddis2016296x3.tif]

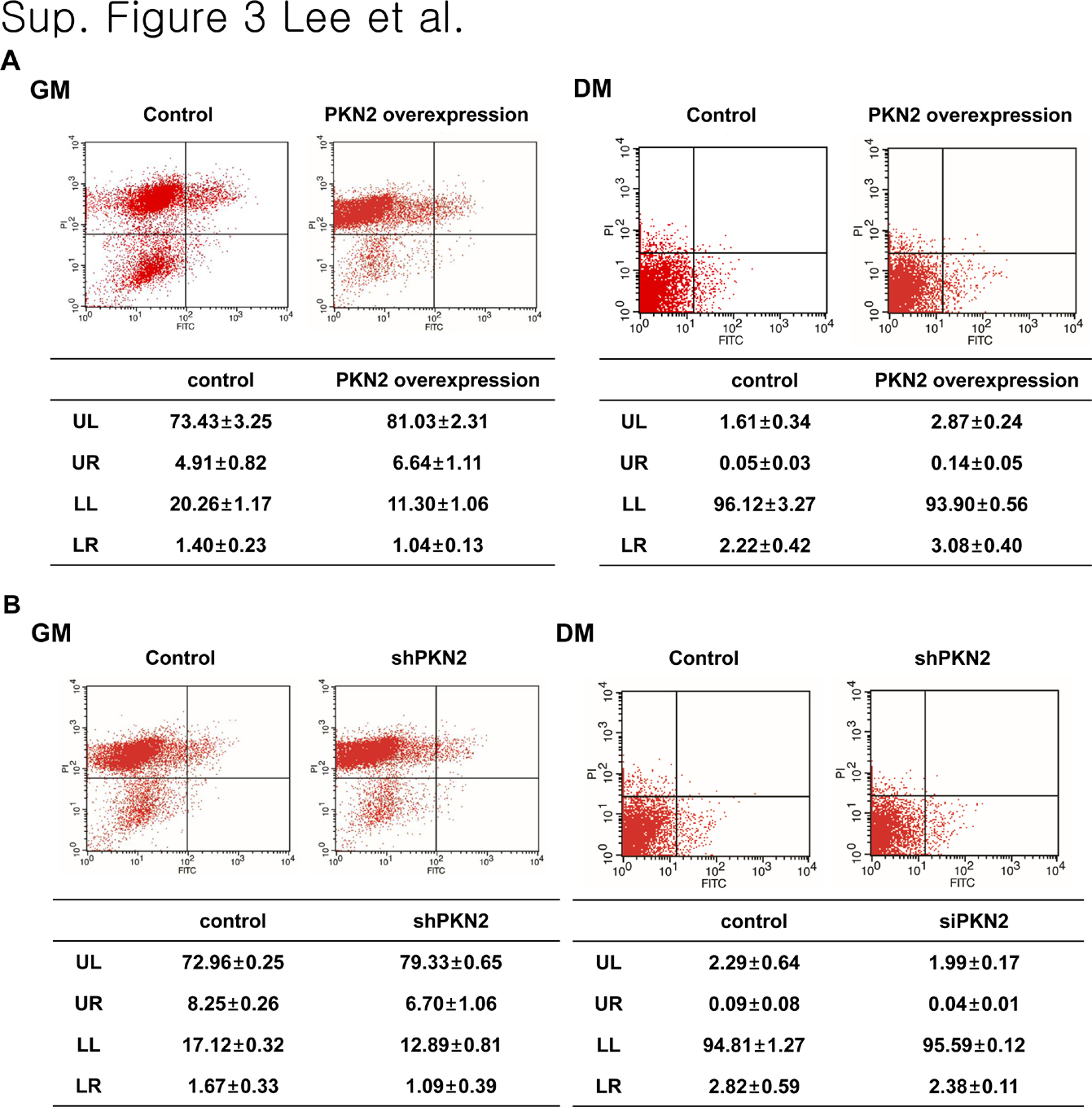

Supplement: Supplementary Figure 3 [file cddis2016296x4.tif]

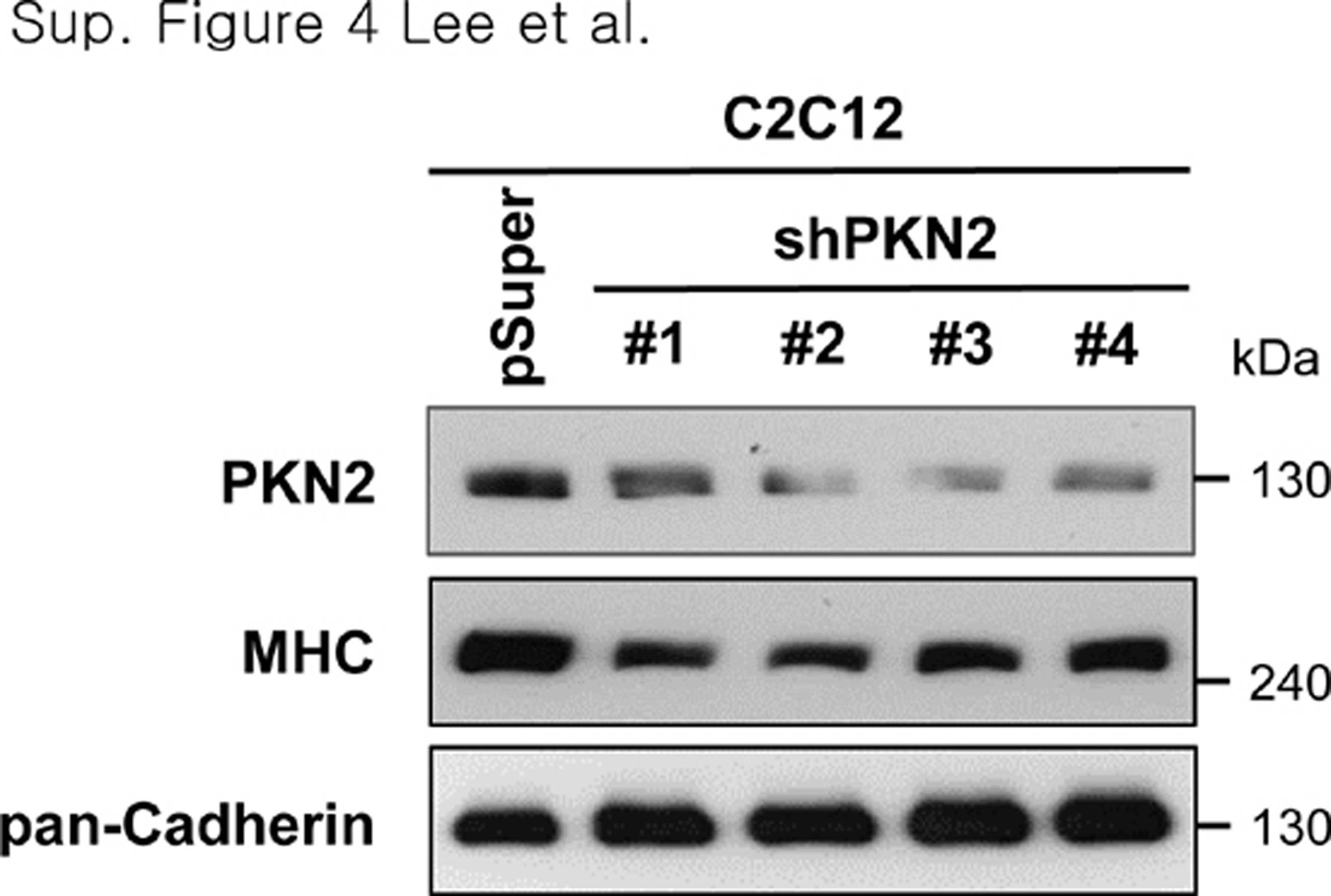

Supplement: Supplementary Figure 4 [file cddis2016296x5.tif]

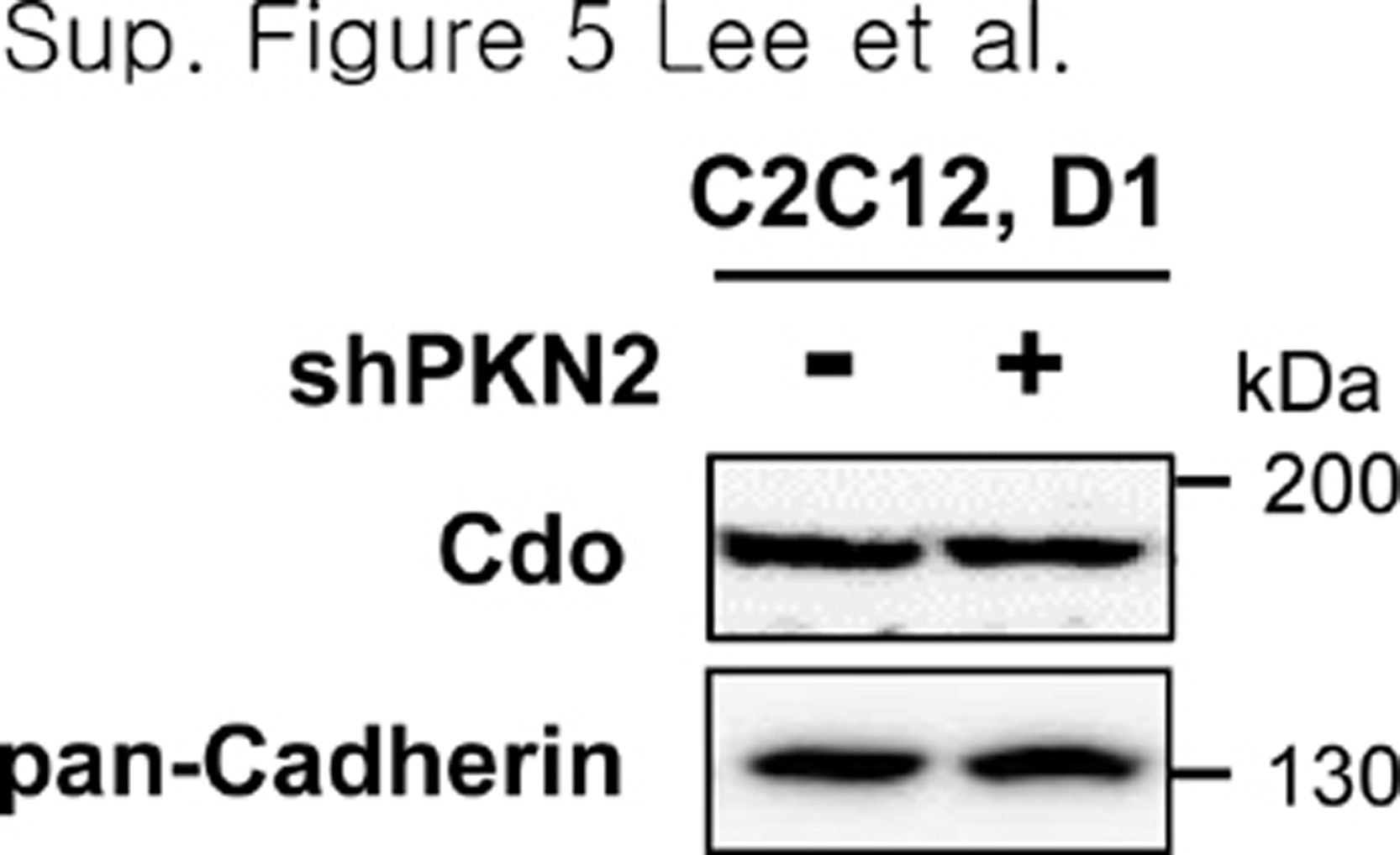

Supplement: Supplementary Figure 5 [file cddis2016296x6.tif]
